# Gaze Palsy as a Manifestation of Todd’s Phenomenon: Case Report and Review of the Literature

**DOI:** 10.3390/brainsci10050298

**Published:** 2020-05-15

**Authors:** Karmele Olaciregui Dague, Manuel Dafotakis, Jörg B. Schulz, Rainer Surges

**Affiliations:** 1 Epileptology Center, Medical Faculty, University Hospital Bonn, Venusberg-Campus 1, 53127 Bonn, Germany; rainer.surges@ukbonn.de; 2 Department of Neurology, University Hospital RWTH Aachen, Pauwelsstraße 30, 52074 Aachen, Germany; mdafotakis@ukaachen.de (M.D.); jschulz@ukaachen.de (J.B.S.); 3 JARA-BRAIN Institute Molecular Neuroscience and Neuroimaging, Forschungszentrum Jülich GmbH and RWTH Aachen University, 52074 Aachen, Germany

**Keywords:** seizure, Todd’s phenomenon, postictal deficits

## Abstract

**Background**: Though Todd’s phenomenon (TP) is a relatively rare occurrence, its correct identification is of key diagnostic and therapeutic importance as a stroke mimic. Here we describe a case of isolated gaze palsy as a manifestation of TP, discuss periictal gaze abnormalities as lateralizing sign involving the frontal eye field (FEF), and present a narrative literature review. **Methods**: We reviewed the main features of the case and conducted a structured literature search of TP and gaze palsy using PubMed. We restricted the search to publications in English, Spanish, French, and German. **Case presentation**: A 71-year-old male with a history of right frontotemporal subarachnoid hemorrhage was admitted to the Emergency Department of our institution after suffering a first unprovoked focal to bilateral tonic-clonic seizure with ictal gaze deviation to the left. Cranial imaging showed no signs of ischemia, intracerebral hemorrhage, or tumor. The patient presented the following postictal features: involuntary eye deviation to the right due to left-sided gaze palsy and disorientation in time with preserved responsiveness. Eye movements were normal three days later. We concluded that the patient suffered from new-onset epilepsy due to sequelae following the right frontotemporal subarachnoid hemorrhage, affecting the FEF with contralateral ictal gaze deviation, and postictal gaze palsy with ipsilateral eye deviation as an unusual Todd’s phenomenon. **Conclusion**: Unusual manifestations of TP are uncommon but clinically highly relevant, as they can mimic stroke or epileptic status and are decisive in the diagnostic and therapeutic decision-making process. Though postictal gaze palsy has been reported associated with other deficits, this constitutes, to our knowledge, the first report of isolated gaze palsy as a form of TP. Further research into the underlying causes is needed. Ictal contralateral gaze and head deviation, and probably postictal ipsilateral gaze deviation if present, are very helpful for the lateralization of the seizure-onset zone.

## 1. Introduction

Though postictal neurological deficits have long been recognized, its underlying causes are not completely understood. A wide variety of neurologic and psychiatric symptoms have been described, posing an important diagnostic challenge, especially to emergency physicians. Here, we describe a case of isolated gaze palsy as an unusual manifestation of Todd’s phenomenon and provide a narrative literature review on postictal signs and the assumed underlying pathophysiology.

## 2. Materials and Methods

After a search using PubMed (search terms “Postepileptic paralysis“, “Todd’s paralysis”, “Todd’s paresis”, “Postictal paralysis”, “Todd’s phenomenon”, “Postictal gaze palsy” “Gaze palsy” and “Postictal hemiplegia”) on February 5, 2018, we carried out a literature review with focus on case reports. We reviewed all available recorded patient characteristics and documentation. 

## 3. Case Report

**Figure 1:**
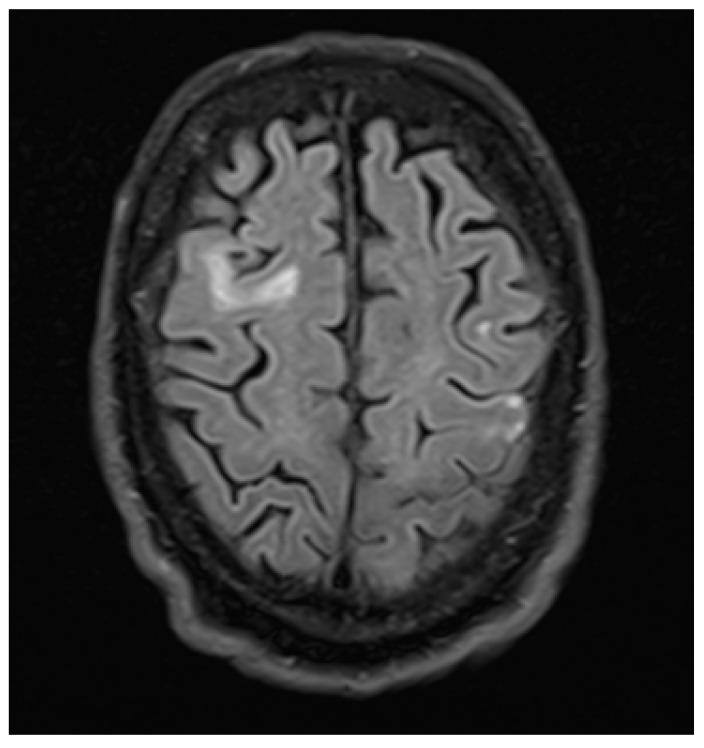
FLAIR cranial MRI image of the postoperative defect of the right FEF.

**Figure 2:**
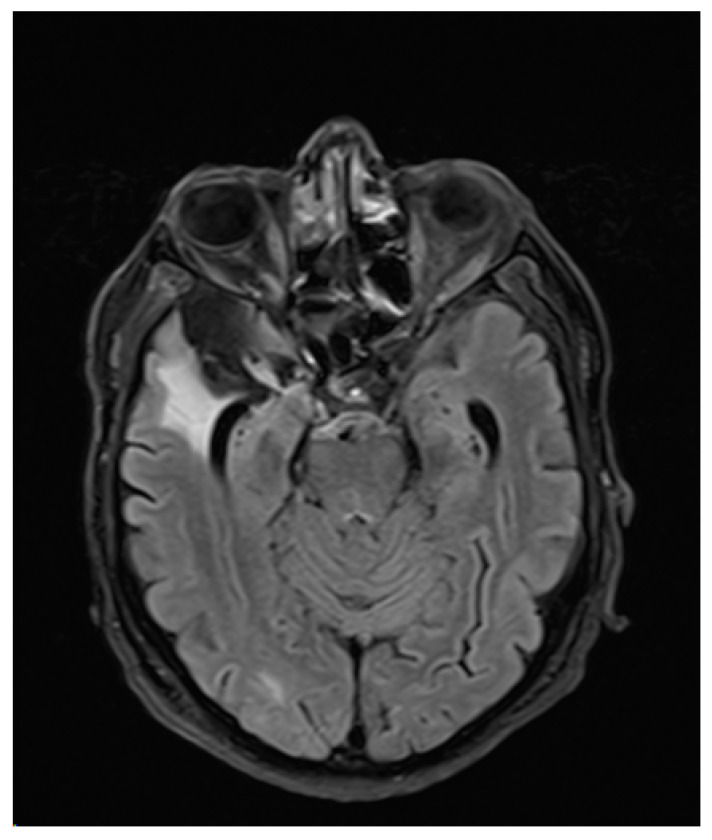
FLAIR cranial MRI image of the parenchymal defect in the right frontobasal region as a result of subarachnoid hemorrhage.

A 71-year old right handed male with a history of oropharyngeal carcinoma and right frontotemporal subarachnoid hemorrhage was admitted to the Emergency Department of our institution after suffering a first unprovoked witnessed focal to bilateral tonic-clonic seizure with forced ictal gaze deviation to the left. Upon arrival, the patient presented the following abnormalities in the neurological examination: slowed psychomotor response, disorientation in space, time, and situation. Hemiparesis, hyperreflexia, sensory deficit, visual field deficit, or nystagmus were not present. Native cranial CT-imaging was carried out shortly after admission and showed right mesiotemporal and frontotemporal substance defects including the right frontal eye field (FEF) following the right frontotemporal subarachnoid hemorrhage and subsequent aneurysm-clipping. No other acute abnormalities were revealed. Initial bloodwork showed slight leucocytosis (12.6/nL) paired with CRP increase (126 mg/L), elevated glucose levels (214 mg/dL), and gamma-GT (99 U/L) as well as elevated TSH (10.16 mU/L). Further examinations (including chest x-ray, urinalysis) showed no signs of infection. Follow-up bloodwork showed normalized leucocyte levels and progressively declining CRP levels. The patient showed no clinical signs of infection. A follow-up cranial CT with arterial angiography revealed no new results. Three days after admission, routine-EEG revealed intermittent dysfunction that was particularly severe in the right frontotemporal region, where localized delta-theta slowing (3–5/s) was apparent, with additional bifrontal delta slowing (2–3/s). Two days later, another routine-EEG showed similar results. After initial therapy with levetiracetam the patient suffered a focal to bilateral tonic-clonic seizure with the following postictal features: involuntary eye deviation (without involuntary head deviation) to the right as sign of left-sided gaze palsy and disorientation in time with preserved responsiveness. Hemiparesis, nystagmus, sensory deficits, and visual deficits were absent. The patient was oriented in time, space, and situation within the following 48 h. The isolated gaze palsy was present for three days, and was initially apparent through manifest involuntary contralateral gaze deviation. During those approximately 72 h, the intensity of the involuntary contralateral gaze deviation progressively declined as the patient’s eye movements returned to normal, until the gaze palsy to the left was only evident during the neurological examination. Four days after admission, a cranial MRI showed no signs of ischemia, intracerebral hemorrhage, or cerebral tumor (Figure 1 and Figure 2). We concluded that the patient suffered from new-onset epilepsy due to sequelae following the right frontotemporal subarachnoid hemorrhage and subsequent aneurysm clipping affecting the right FEF, with contralateral ictal gaze deviation and isolated postictal gaze palsy with ipsilateral eye deviation as an unusual Todd’s phenomenon. We found no other cases of isolated gaze palsy as TP in the literature.

## 4. Discussion

The term “Gaze Palsy” describes an impairment of conjugate eye movements, the movements of both eyes in the same direction. These include saccades, optokinetic, and vestibulo-ocular responses, among others. Unilateral deficits and deficits of convergence therefore do not fall under this descriptor, although they impair gaze [1]. Due to the divergence of the prenuclear control pathways for vertical and horizontal eye movement, in most cases gaze palsies occur in one axis only. The oculocephalic reflex relies on the infranuclear systems and is therefore preserved in cases of horizontal gaze palsy. Contrary to the impairments due to lesions of the frontal eye field (FEF), brainstem lesions that lead to horizontal gaze palsy cause deficits of eye movements ipsilateral to the lesion. In nuclear and infranuclear pontine lesions, the oculocephalic reflex is also abolished [2].

The frontal eye fields (FEF) are located in Brodmann’s area 8 in both hemispheres. Intracranial stimulation studies dating back to Wilder Penfield’s pioneering research [3] showed that cortical electric stimulation of the FEF provokes contralateral conjugated eye movement, which may precede head deviation to the contralateral side [4].

While gaze palsies are most frequently caused by stroke with subsequent eye deviation ipsilateral to the acute lesion, transitory deviation of the eyes and head is not seldom observed during seizures involving the FEF with gaze deviation contralateral to the seizure-onset zone. Indeed, previous works show that head and eye deviation is an excellent lateralizing sign, with more than 90% of cases manifesting as deviation contralateral to the frontal lobe seizure lesion a consequence of epileptic activity within the FEF [5,6]. Postictally, the deviation may reverse as a manifestation of TP, most probably because of the relatively increased input of the unaltered FEF of the opposite hemisphere [7]. 

Postictal deficits were first described by Louis-François Bravais as “l’épilepsie hémiplégique” in 1827 [8], and later, in 1849 by Robert Bentley Todd, whom the current eponym honors, as “epileptic hemiplegia” [9]. Though its underlying mechanism is still unclear, several theories have been proposed [10], some by Todd’s disciples William Gowers (namesake of “Gower’s sign”) and John H. Jackson (of the eponym “Jacksonian march”). Gowers suggested an inhibition of the cortical tissue involved in the seizure as a form of compensation [11], while Jackson postulated an exhaustion of said tissue after ictal activity [12]. More recent works referred to elevated lactate levels as the mechanism behind the aforementioned exhaustion [13]. Other publications point to a possible cerebrovascular origin, as changes in perfusion or arteriovenous shunting [14,15]. Hyperperfusion has long been reported during ictal activity, in CT perfusion imaging [16], as well as in single positron emission tomography (SPECT) imaging in patients with temporal lobe epilepsy [17]. SPECT findings in the postictal [18] and interictal [17] state, however, present hypoperfusion. Several case reports of TP have shown postictal local cortical hypoperfusion in CT as well as MRI perfusion imaging [19,20], though localized postictal hyperperfusion in CT perfusion imaging has also been reported [21]. As a possible explanation for the inconsistency in the reported findings, imaging tests may have been performed at different stages of the pathophysiological timeline leading from ictal to postictal state, showing different degrees of cortical perfusion. Unfortunately, we were not able to carry out CT-perfusion imaging during our patient’s isolated postictal gaze palsy. CT-perfusion imaging after the initial seizure without postictal deficits showed no pathological findings. 

Farrell et al. [22] observed that postictal paresis is due to sustained cerebral hypoperfusion in the postictal state. Their data suggest that bilateral tonic-clonic seizures (BTCS) and/or postictal deficits may have lasting effects, as they detected severe local hypoxic conditions in rodents as a consequence of hypoperfusion for approximately one hour after BTCS and were able to reproduce these findings in a small group of patients. Moreover, they were able to reduce hypoxia in rodent models by inhibiting cyclooxygenase-2 or L-type calcium channels, opening a possible line of research into therapeutic interventions in human patients. Interestingly, Rolak et al. [23] showed 57% of patients with TP in their study population of white male veterans had an underlying structural lesion, namely ischemic stroke of the middle cerebral artery. Furthermore, Gallmetzer et al. reported hippocampal sclerosis or atrophy in 38.6% of cases, and other lesions in 29.5% [24]. This may suggest that pre-damaged areas of the brain are more susceptible to the pathophysiological changes that lead to Todd’s phenomenon, and could mean patients with symptomatic epilepsy are predisposed to postictal deficits. 

Since Bravais’ and Todd’s publication of postictal hemiplegia, several different postictal neurologic and psychiatric symptoms have been reported, namely hemineglect and apraxia [25], mydriasis [26], sensory deficits [23], hemianopsia [27], blindness [28,29,30], mutism [31], confusion [32], bulimia [33], dyscalculia and deficits of visuospatial perception [34], ataxic hemiparesis [35], aphasia [36] and one case of fire-setting behavior [37]. Gaze palsy was reported in 3 of 7 patients in a case series, accompanied by aphasia and/or hemiplegia and hemianopsia [38]. Furthermore, postictal psychosis appears to be rather frequent and occurs in 2–6% of patients [39,40]. Interestingly, TP has also been described after general anesthesia [41] and after interscalene block [42].

We found no other cases of isolated gaze palsy as TP in the literature. Case reports and their main clinical characteristics are listed in Table 1. Postictal gaze palsy remains a rare finding, and when it indeed occurs, has been reported to be accompanied by other deficits. This case of gaze palsy is, to the best of our knowledge, the first to be reported as an isolated postictal deficit.

Though there are numerous case reports describing varied manifestations of TP, there are few studies that systematically analyze its occurrence, frequency and causes. 

Duration of postictal deficits has been reported with a wide range of variation. A video-EEG study [24] documented deficits lasting 11 seconds to one hour, while other works referred that deficits persisted from 30 minutes to 36 hours [23], and deficits lasting up to 6 weeks have been documented [17]. Regarding the frequency of Todd’s phenomenon, reports from video-EEG studies [36] vary. A previous study documented TP in 4 (3.3%) of 60 patients [43], whereas another case series described TP in 44 (14.3%) of 513 patients [24]. Data published in the latter study did not support a relationship between location of the seizure onset zone and frequency and duration of deficits [24]. While some works did not find a relationship between characteristics of the seizures that led to TP and the features of the postictal deficits themselves [23], others found clonic ictal activity correlated with longer postictal paresis [24]. Occurrence of TP was not reported to be more frequent in temporal or extratemporal seizure onset zones [24]. 

In regard to EEG findings, several video-EEG studies specifically reported alterations related to TP. Both described slow (theta or delta) contralateral activity during seizures in several patients with TP, in some cases with additional epileptiform discharges [23]. Among the patients who suffered TP during EEG recording, one was normal [23], and one showed slow contralateral activity [43]. Though we were not able to carry out EEG during our patient’s postictal isolated gaze palsy, our EEG findings are consistent with this pattern of slow activity in the epileptogenic area as well as the contralateral counterpart. All published works included in this review emphasize the excellent lateralizing value of TP, which may be clinical use when defining the seizure-onset zone.

## 5. Conclusions

Currently, published data suggests TP is a cerebrovascular phenomenon. Imaging studies investigating Todd’s phenomenon seem sometimes to be contradictory, though the timing of imaging may be a decisive factor to draw conclusions from these data. Duration of postictal deficits varies from seconds to weeks in published reports. Published data suggest that pre-damaged areas of the brain are more susceptible to the pathophysiological changes that lead to Todd’s phenomenon. Some studies found clonic ictal activity correlated with longer postictal paresis. Published data do not support a relationship between location of the seizure onset zone and frequency and duration of deficits. Unusual manifestations of TP are uncommon but clinically highly relevant, as they can mimic stroke or epileptic status and are decisive in the diagnostic and therapeutic decision-making process. Though postictal gaze palsy has been reported associated with other deficits, this constitutes, to our knowledge, the first report of isolated gaze palsy as a form of TP. Further research into the underlying causes of TP is needed. Ictal contralateral gaze and head deviation, and probably postictal ipsilateral gaze deviation if present, are very helpful for the lateralization of the seizure-onset zone.

## Figures and Tables

**Table 1:** Case report characteristics.

Reference Number	Postictal Neurological Deficit	Duration	Type of Epilepsy	Seizure Type Prior to Onset of TP Symptoms	EEG Findings during TP	Imaging
**25**	Apraxia, Hemineglect	72 h	TLE	BTCS	No	MRI: No epileptogenic lesions
**27**	Hemianopsia	1 month	Symptomatic	BTCS	No	MRI: Glioblastoma multiforme
**28**	Blindness	Up to 3 days	N/A	N/A	N/A	N/A
**31**	Mutism, right hemiparesis	48 h	N/A	BTCS series	No	CT: normal
**32**	Confusion	4–10 days	N/A	Focal and generalized	Typical encephalopathic pattern	9 of 11: Minimal structural abnormalities
**34**	Neglect, dyscalculia, and disturbed visuospatial perception	1 month	Symptomatic parietal lobe epilepsy	Convulsive status epilepticus	Diffuse amplitude reduction in the right hemisphere	MRI: Pachygyria and polymicrogyria in the right parietal cortex
**35**	Ataxic hemiparesis	7 days	N/A	N/A	N/A	N/A
**29**	Blindness	N/A	Focal epilepsy	Focal posterior cingulate gyrus seizure	N/A	N/A
**38**	Hemiplegia, global aphasia, gaze palsy	2 days	Symptomatic epilepsy with focal and focal to bilateral tonic-clonic seizures	Convulsive status epilepticus	N/A	Postischemic defects, localization N/A
	Lower limb paralysis, global aphasia, cognitive disorder	2 months	First onset	Convulsive status epilepticus	N/A	Postischemic defects, meningeomas, localization N/A
	Hemiplegia, sensory aphasia	2 months	Symptomatic epilepsy	Focal to bilateral tonic-clonic seizure	N/A	Postischemic defects, localization N/A
	Quadriplegia, motor aphasia, gaze palsy, cognitive disorder	3 months	Symptomatic epilepsy	Convulsive status epilepticus	N/A	Postischemic and posthemorrhagic defects, localization N/A
	Lower limb paralysis, global aphasia, cognitive disorder	3 months	First onset	Convulsive status epilepticus	N/A	MRI: Bilateral reversible white matter damage CT Perfusion imaging: prolonged bilateral cortical mean transit time, cortical cerebral blood volume reduced
	Hemiplegia, global aphasia, disorder of consciousness	2 months	First onset	Convulsive status epilepticus	N/A	Postischemic defects, localization N/A
	Global aphasia, gaze palsy, hemianopsia	2 days	N/A	Convulsive status epilepticus	N/A	N/A

Abbreviations: BTCS: Bilateral tonic-clonic seizure; N/A: not available; TLE: temporal lobe epilepsy.
